# A Novel Strategy for Live Detection of Viral Infection in *Drosophila melanogaster*

**DOI:** 10.1038/srep26250

**Published:** 2016-05-18

**Authors:** Jens-Ola Ekström, Dan Hultmark

**Affiliations:** 1 Department of Molecular Biology, Umeå University, S-90187 Umeå, Sweden; 2 BioMediTech, FI-33014 University of Tampere, Finland

## Abstract

We have created a transgenic reporter for virus infection, and used it to study Nora virus infection in *Drosophila melanogaster*. The transgenic construct, *Munin*, expresses the yeast transcription factor Gal4, tethered to a transmembrane anchor via a linker that can be cleaved by a viral protease. In infected cells, liberated Gal4 will then transcribe any gene that is linked to a promoter with a UAS motif, the target for Gal4 transcription. For instance, infected cells will glow red in the offspring of a cross between the *Munin* stock and flies with a *UAS-RFP*^*nls*^ transgene (expressing a red fluorescent protein). In such flies we show that after natural infection, via the faecal-oral route, 5**–**15% of the midgut cells are infected, but there is little if any infection elsewhere. By contrast, we can detect infection in many other tissues after injection of virus into the body cavity. The same principle could be applied for other viruses and it could also be used to express or suppress any gene of interest in infected cells.

Nora virus is a currently unclassified picorna-like virus that naturally infects the fruit fly *Drosophila melanogaster*^1^. This virus causes persistent infection with no obvious symptoms or pathogenesis. Like many other picorna-like viruses, it is spread by faecal-oral transmission^2^. A striking characteristic of the Nora virus infection is that the virus titre differs enormously between individual fruit flies that are kept in the same vial and the titres may vary from 10^2^ up to 10^10^ viral genomes per fly^2^,^3^. We study Nora virus as an animal model for persistent virus infection.

Virus detection is crucial in all virus research and several methods have been used for this purpose. Viruses can be detected by their specific pathogenesis in human and animal models, changes in morphology and viability of cultured cells, detection of viral nucleic acid or use of antibodies and other molecular markers^4^,^5^,^6^,^7^,^8^,^9^,^10^. The Nora virus does not cause obvious symptoms and there are no antibodies available for immunohistochemical labelling. This encouraged us to develop a new molecular technique to facilitate viral studies in the fruit fly. Besides detecting the mere presence of viruses, this technique permits virus-triggered expression of any gene of choice specifically in virus-infected cells. This can be used to express a molecular marker for virus detection or, for example, for targeted expression or suppression of genes involved in the host’s viral immunity.

Several techniques are developed to modify gene expression in *Drosophila melanogaster*, for example the popular Gal4-UAS system^11^. Transgenic fruit flies can express the transcription factor Gal4 from the yeast *Saccharomyces cerevisiae*, which acts on a yeast-specific promoter motif, the upstream activating sequence (UAS). Tens of thousands of such “driver” fly stocks are available for the Gal4-UAS expression system, which in a combinatorial way allow expression of virtually any gene in any tissue. A common application is to express an RNA hairpin that feeds into the fruit fly’s RNA interference (RNAi) pathway, in order to suppress the expression of an endogenous *Drosophila* gene.

We designed a Gal4-UAS-based expression system that depends on the activity of the Nora virus-encoded protease. For this purpose we constructed a *Drosophila* driver stock with constitutive and ubiquitous expression of a target to the viral protease. The target is the Gal4 protein fused to a transmembrane anchor, via a linker that has a cleavage site for the virus-encoded protease (Fig. 1A). This anchors Gal4 in the cytoplasmic space, preventing this transcription factor from entering the nucleus and from initiating transcription from UAS promoters. Upon virus infection the linker is cleaved by the virus protease, allowing Gal4 to enter the nucleus and initiate gene expression. Like in the system reported recently by Ren *et al.*^10^ this amplifies a signal created by the action of a virus-specific protease. In addition, the system described here will permit expression of any gene of the researcher’s choice specifically in virus-infected cells.

We call this virus protease-dependent gene expression system Munin. As an application we used it to drive red fluorescent protein expression for *in vivo* detection of Nora virus in the fruit fly. This reporter system allowed us to determine which tissues the Nora virus naturally infects.

## Results and Discussion

**Figure 1:**
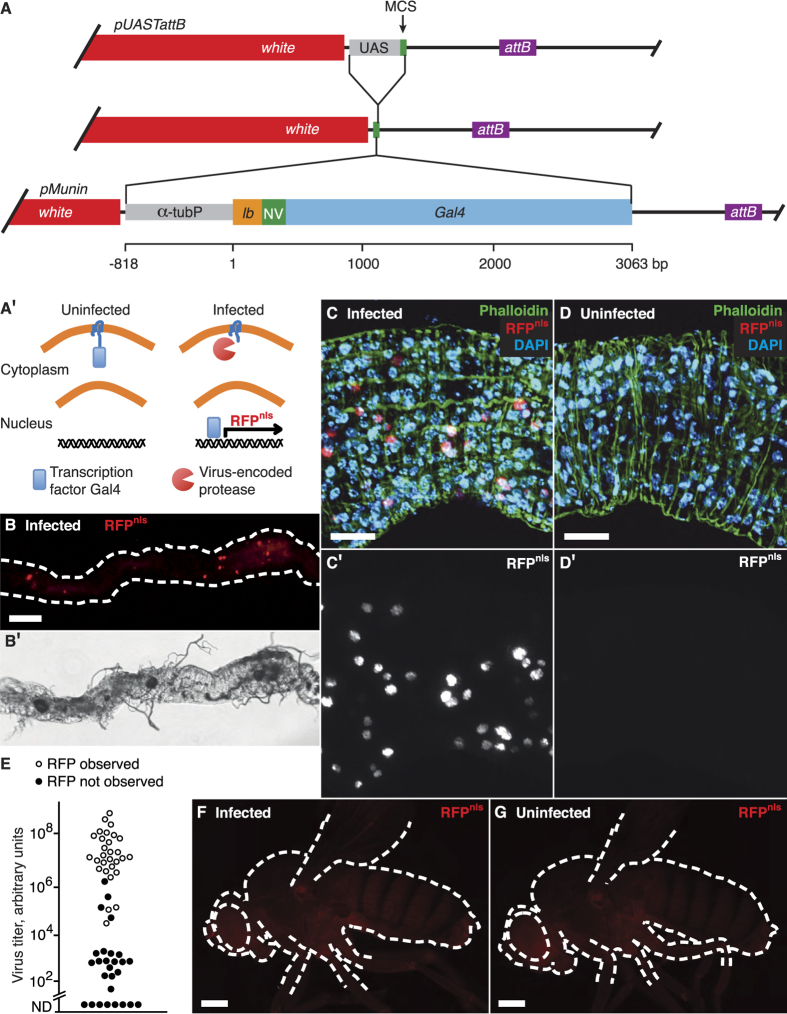
Nora virus replication in flies infected by oral ingestion. (**A**, **A**′) Construction and molecular mechanism for the Munin virus reporter. (**A**) The transformation vector pUASTattB^21^ was adapted to constitutively drive expression of the inserted gene and the Munin reporter construct was inserted. (**A**′) The Munin reporter is ubiquitously expressed in the fly but the transcription factor Gal4 can enter nucleus only when there is an active virus-encoded protease in the cytoplasm. MCS, multiple cloning site. α–tubP, α-tubulin 84B promoter. *lb*, *late bloomer* transmembrane region. NV, Nora virus protease target site. (**B–D**) RFP^nls^ expression in the Nora virus-infected midgut. C and D are post-processed to enhance the red fluorescence. (**E**) Correlation between RFP^nls^ expression and the amount of Nora virus RNA excreted in the faeces, as determined by quantitative RT-PCR. Each data point represents a single fly. The graph shows the results of 56 flies from three independent experiments. An additional 16 low-titre flies were not scored for fluorescence. ND, not detected. (**F**,**G**) No obvious difference between infected and uninfected whole flies when observed from the outside. Infection was confirmed in F by a detected high virus titre in the faeces. Scale bars show 100 μm in (**B**), 25 μm in (**C**,**D**) and 250 μm in (**E**–**G**).

The Munin reporter construct contains a tetraspanin transmembrane anchor, fused to Gal4 via a Nora virus protease cleavage motif (Fig. 1A,A′). The construct was inserted into a *Drosophila* P element vector and transgenic fly lines were generated. The *w*; *UAS-RFP*^*nls*^; *Munin* reporter fly stock also expresses red fluorescent protein tagged with a nuclear localisation signal (RFP^nls^), under the control of the Munin reporter. To test the Munin reporter we established a persistent Nora virus infection in the fly stock. The flies were thus environmentally exposed to the virus via faeces deposited in the fly vial and they ingested the virus with the food. We dissected adult flies one week after eclosion and examined their abdominal organs for RFP^nls^ expression by fluorescence microscopy. Our previous results suggested that Nora virus is an enteric virus^2^ and in line with this we observed about 20 cells in the midgut with strong red fluorescence (Fig. 1B). Enhancing the red fluorescence channel by image processing revealed a large number of more weakly expressing cells. Thus, RFP^nls^ expression varies considerably between the infected cells (Fig. 1C), possibly indicating progressive infection of previously uninfected cells. In total we find that 5–15% of the midgut cells expressed RFP^nls^.

A characteristic of the Nora virus infection is that the virus titres differ several orders of magnitude between the individuals in an infected fly stock. This is reflected by highly variable amounts of viral RNA excreted in the faeces^3^. To identify high-titre individuals we determined the amount of Nora virus genomic RNA in the faeces by quantitative RT-PCR. Blinded from the viral quantities we also examined the abdominal organs for RFP^nls^ expression. We observed RFP^nls^ expression in the midgut in all flies with a high virus titre and in some flies with an intermediate titre, but no RFP^nls^ was observed in flies with a low virus titre (Fig. 1C) or in uninfected flies (Fig. 1D). The quantification is shown in Fig. 1E. We conclude that the Munin reporter is a reliable reporter of Nora virus infection. The low levels of Nora virus RNA in the low-titre flies probably originate from virus that has been ingested with the food without producing an active infection in the animal. We have previously shown that the virus can be cleared from such low-titre animals by serial transfer of the flies to fresh uncontaminated food^2^, as expected if no cells are infected. Thus, expression of the Munin reporter is tightly correlated with the presence of a productive Nora virus infection.

To test the general usefulness of the Munin reporter design, we constructed a corresponding reporter fly stock for the *Drosophila* C virus. When injected with the C virus, the flies showed classical signs of disease by swelled abdomen and death after a few days. C virus-infected flies also showed obvious RFP expression in their abdomen, whereas uninfected flies did not express RFP (Supplementary Fig. S1). Importantly, C virus reporter flies injected with Nora virus did not express RFP two weeks after the viral infection. This further supports virus-specific expression by the Munin reporter and it also suggests that the reporter can be designed to recognise only a certain group of virus.

**Figure 2:**
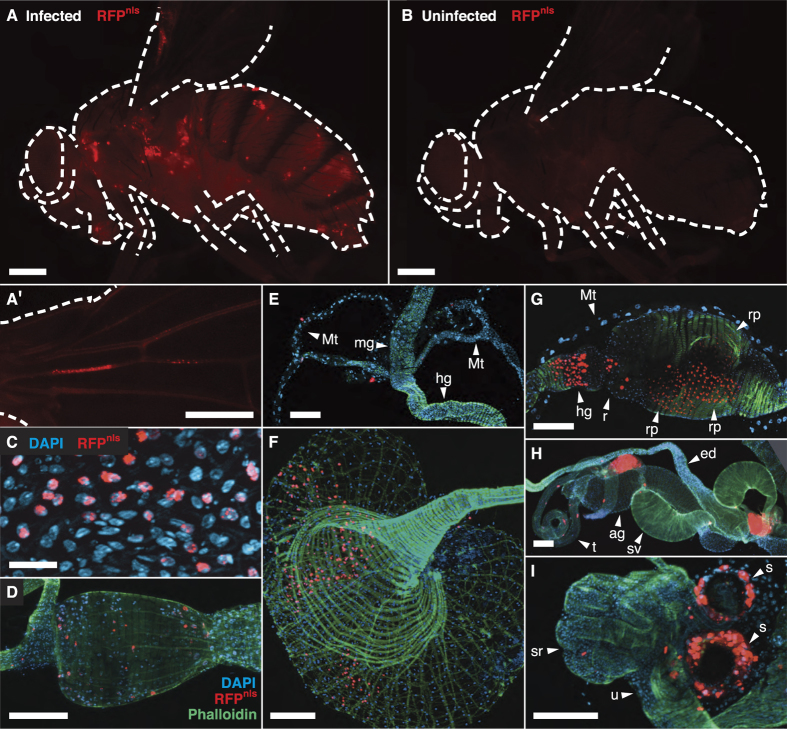
Nora virus replication in flies infected by injection. (**A**–**D**) All injected animals showed RFP^nls^ expression in epidermis, cardia and midgut. (**A**) RFP^nls^ was expressed in a spotted pattern in epidermal cells. In addition, stray light from fluorescing cells lights up the whole body, except where it is pigmented. (A′) RFP^nls^ expression in the wing veins. (**B**) Only faint autofluorescence was seen in uninfected flies, at the same level as in wild-type control flies. (**C**) Midgut. Only enterocytes (cells with a big nucleus) expressed RFP^nls^. (**D**) Cardia. (**E**–**I**) In rare cases crop, Malpighian tubules, hindgut, rectum and parts of the reproductive tract expressed RFP^nls^. (**E**) Malpighian tubules (Mt), here shown on each side of midgut (mg) and hindgut (hg). (**F**) Crop. (**G**) Hindgut and rectum. The posterior section of hindgut (hg), rectal epithelium (r) and rectal papillae (rp). (**H**) The male reproductive tract. Testis (t), seminal vesicle (sv), accessory gland (ag) and ejaculatory duct (ed). This specimen has strong expression in the proximal end of the testes, just next to the testicular ducts that connect the testes to the seminal vesicles. (**I**) Parts of the female reproductive tract. Uterus (u), seminal receptacle (sr) and spermathecae (s), with strong expression in cells surrounding the melanized cuticle. Scale bars show 250 μm in A,B, 25 μm in C and 100 μm in (**D**–**I**).

**Figure 3:**
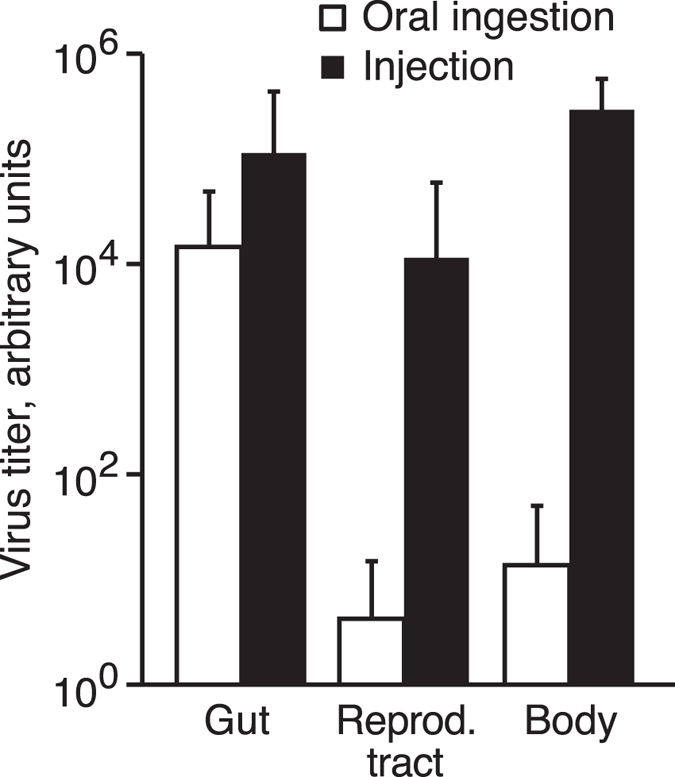
Quantitative RT-PCR for Nora virus RNA in different body parts. Animals infected by oral ingestion have viral RNA essentially only in the gut, whereas animals injected with the virus have a substantial amount of viral RNA in additional tissues. “Body” refers to all tissues except gut and reproductive tract. The flies were four weeks old at the time for the analysis. Error bars show standard deviation. Data are based on two independent experiments involving in total 10 flies in each group. Data for each fly are shown in Supplementary Fig. S2.

The precise labelling of virus-infected cells with the Munin reporter allowed us for the first time to investigate in detail which tissues that are infected by Nora virus. We investigated flies up to four weeks after eclosion, but we never observed RFP^nls^ expression in any other tissues besides the midgut. Four different cell types have been described in the *Drosophila* midgut epithelium. A self-renewing population of intestinal stem cells produces enteroblasts, which differentiate to enterocytes (about 90%) or enteroendocrine cells (about 10%). Enterocytes are morphologically distinguishable from the other cell types by their big nuclei^12^,^13^. We only saw RFP^nls^ in cells with a big nucleus, indicating that Nora virus has tropism for enterocytes (Fig. 1C; see also Fig. 2C). Quantitative RT-PCR for Nora virus RNA in different tissues supports the conclusion that Nora virus only infects the alimentary canal (Fig. 3 and ref. 2). Nora virus RNA was mainly found in the gut, whereas viral RNA concentration in the reproductive tract and other parts of the body was at least a 1000-fold lower (Fig. 3, and Supplementary Fig. S2). Such low concentrations are most likely due to contamination from faeces during dissection of the flies.

As most viral studies performed in *Drosophila* are based on animals that are infected by injection of the virus into the blood, we also investigated Nora virus replication in flies infected this way. In all injected animals we saw RFP^nls^ expressed in epidermis and cardia in addition to the midgut (Fig. 2A–D). In rare cases we also observed RFP^nls^ expression in the crop, Malpighian tubules, the posterior part of hindgut, rectum or the reproductive tract (Fig. 2E–I). For the reproductive tract, our data do not allow characterisation of which specific cells are infected, but this may include sperm heads in the proximal end of the testes, epithelial cells, and secretory cells in the spermathecae (Fig. 2H,I). Quantitative RT-PCR show that injected animals had increased amount of viral RNA in the body and in the reproductive tract (Fig. 3, and Supplementary Fig. S2). Furthermore, all injected flies had a high virus titre and there was no longer a group of animals with low virus titre as seen after oral ingestion (Supplementary Fig. S3). Altogether, we conclude that additional tissues are infected after injection of the virus into the blood. Thus, Nora virus infection by injection causes a radically different outcome compared to infection by oral ingestion.

Although Nora virus has the capacity to infect other tissues in the fruit fly, it remains contained in the midgut after oral ingestion. The gut thus constitutes an efficient barrier that prevents Nora virus spread to other tissues. This could also be the case for other viruses that are naturally transmitted by the faecal-oral route, for example the *Drosophila* C virus. When infecting by oral ingestion, the C virus is usually non-pathogenic^14^, but it causes lethality within a few days after injection into the blood^15^. C virus infection after oral ingestion is surprisingly little studied. The virus replicates in the larval midgut^16^, but to our knowledge it is not yet described which tissues that are infected in the adult fly. In contrast, the pathogenesis after infection by injection is well characterised. The C virus replicates in many different tissues, including the visceral muscles^15^,^17^, causing obstruction of the gut and starvation or dehydration to death^18^. Thus, systemic infection of the C virus has dramatic consequences to the fly. The gut epithelium might therefore play a crucial role in limiting spread of the *Drosophila* C virus, as for the Nora virus. However, in studies of insect viral immunity it is currently common practise to inject the virus into the blood, thereby bypassing the normal infection route. The gut is therefore very little studied for its role in viral immunity.

Our results show that the Munin construct is a useful tool to study Nora virus infection and that the technique can be adapted also to other viruses and it can most likely be adapted to other hosts. We had initially hoped to use the Munin reporter to score viral infection directly in living flies, but this was only possible with animals infected by injection. RFP^nls^ expression is too faint to detect gut infections with our instruments without dissection of the abdomen (Fig. 1F,G). Future developments could be to combine Munin with other UAS-dependent reporters, or to use more sensitive imaging equipment.

## Material and Methods

### Munin reporter, fly strains and fly maintenance

We constructed the Munin reporter gene by inserting the first 216 bp (encoding the first two transmembrane helices) of the *Drosophila* tetraspanin *late bloomer*, followed by 180 bp cDNA sequence of the Nora virus genome encoding the VP4B-VP4C junction protease cleavage site^19^, and the complete 2646 bp Gal4 gene, in a cloning vector. In the corresponding *Drosophila* C virus reporter we inserted a 180 bp cDNA sequence encoding the VP2-VP3 junction protease cleavage site, as determined for the closely related cricket paralysis virus^20^. For constitutive and ubiquitous expression we modified the *pUASTattB* transformation vector^21^ by replacing the UAS promoter with the α-tubulin 84B promoter^22^,^23^, and we inserted the Munin reporter construct in the new transformation vector. We call the final construct *pMunin*. The complete sequence of the final transformation plasmid is available in the Supplementary Appendix. The Munin reporter was transformed into *y*^1^, *w*^1118^; PBac (*y*+−attP-9A) VK00013 flies, which have a predestined landing site at 3L:19197458^24^. Transformation was performed by BestGene Inc., USA.

A *w*^1118^; P{*w*[+mC] = UAS-RedStinger}4/CyO stock^25^ was obtained from the Bloomington Drosophila Stock Center. We refer to RedStinger as RFP^nls^ in this work. The flies were reared on standard mashed potato-agar fly food^26^ and kept in 25 °C and 60% relative humidity. All fly stocks were treated with Tetracycline as described^27^ to remove potential *Wolbachia* infections.

### Virus and virus infection procedures

*Drosophila* Nora virus strain Umea 2007 was derived from an infectious cDNA clone^19^ (accession GQ257737) and had been continuously passaged in *w*; +; *Relish*^*E23*^ flies^28^ for more than two years before performing the experiments described in this work.

Flies were infected by environmental exposure to the virus via contamination of the food by the faeces of their virus-infected parents, or by microinjection of a virus-containing suspension into the blood. For the latter, Nora virus suspension was prepared by homogenising approximately 100 infected *w*; +; *Relish*^*E23*^ flies in 0.5 ml PBS and filtering the suspension through a 0.45 μm filter. Using an Inject + matic microinjector (Inject + matic, Geneva, Switzerland), we injected approximately 100 nl of the virus suspension into the thorax of each fly via the thin cuticle under the wing hinge. Infected this way, the flies show an 100–1000 fold increase of Nora virus RNA within ten days after injection^2^. For environmental exposure, we established a persistently infected fly stock by injecting the virus into flies, and we used their progeny in the experiments.

We used a *Drosophila* C virus strain of uncertain origin that has been kept in our lab. The region around the protease cleavage site was sequenced (see Supplementary Appendix) and found 97% identical to *Drosophila* C virus strain EB (accession AF014388). The virus was propagated in *Drosophila* S2 cells until 50% of the cells had lysed. The suspension was freeze-thawed three times to completely lyse the cells, diluted 10^5^ times in phosphate-buffered saline, pH 7.5 (KCl 0.2 g/l, KH_2_PO_4_ 0.2 g/l, NaCl 8 g/l, Na_2_HPO_4_ anhyd. 1.15 g/l), and filtered through a 0.22 μm filter before injection into flies as described above.

### Nora virus quantification

Nora virus titres were determined by one-step quantitative RT-PCR as described^2^,^19^. For the amount of Nora virus in the faeces, we kept individual flies in clean fly vials and we exchanged to clean vials once per day. On the third day we transferred the flies to 1.5 ml centrifuge tubes with 10–20 μg fly food, and the flies were kept in these tubes for 18 h. The viral RNA deposited with faeces was collected by vortexing the tubes with Total RNA Lysis Solution provided in the Aurum Total RNA Mini Kit (Bio-Rad).

### Microscopy

Dissected organs were fixed in 3.5% paraformaldehyde for 10 min at room temperature, permeabilised with 0.1% Triton X-100 for 10 min, incubated in CytoPainter Phalloidin iFlour 488 (Abcam) for 30 min, and mounted in ProLong Gold antifade reagent with DAPI (Life Technologies). Images were acquired in Zeiss ApoTome.2 and Zeiss Lumar.V12 microscopes, and the contrast was enhanced with the Adobe Photoshop software. Images of control and infected animals were always processed the same way. Contrast was equally enhanced in Figs 1 and 3.

## Etymology

In the Norse mythology, Hugin and Munin is a pair of ravens that fly over the worlds every day. They see everything that happens and they bring all news to the god Odin, who is thereby kept informed^29^.

## Additional Information

**How to cite this article**: Ekström, J.-O. and Hultmark, D. A Novel Strategy for Live Detection of Viral Infection in *Drosophila melanogaster*. *Sci. Rep.* **6**, 26250; doi: 10.1038/srep26250 (2016).

## Supplementary Material

Supplementary Information
