# Supplementary material for: A Novel Strategy for Live Detection of Viral Infection in Drosophila melanogaster
Source: Sci Rep. 2016 May 18;6:26250. doi: 10.1038/srep26250 (PMC4870574; doi:10.1038/srep26250)
Supplement: Supplementary Information [file srep26250-s1.pdf]

Supplementary Information:

A Novel Strategy for Live Detection of Viral Infection in *Drosophila melanogaster*

Jens-Ola Ekström[1,2] and Dan Hultmark[1,2]

Contents

Figure S1	p. 1
Figure S2	p. 2
Figure S3	p. 2
Appendix	
Complete sequence of the Nora virus Munin reporter transformation plasmid.	p. 3
Unique part of the C virus reporter sequence.	p. 6

[1]Department of Molecular Biology, Umeå University, S-90187 Umeå, Sweden. [2]BioMediTech, FI-33014 University of Tampere, Finland. Correspondence and requests for materials should be addressed to D.H. (email: dan.hultmark@ucmp.umu.se)

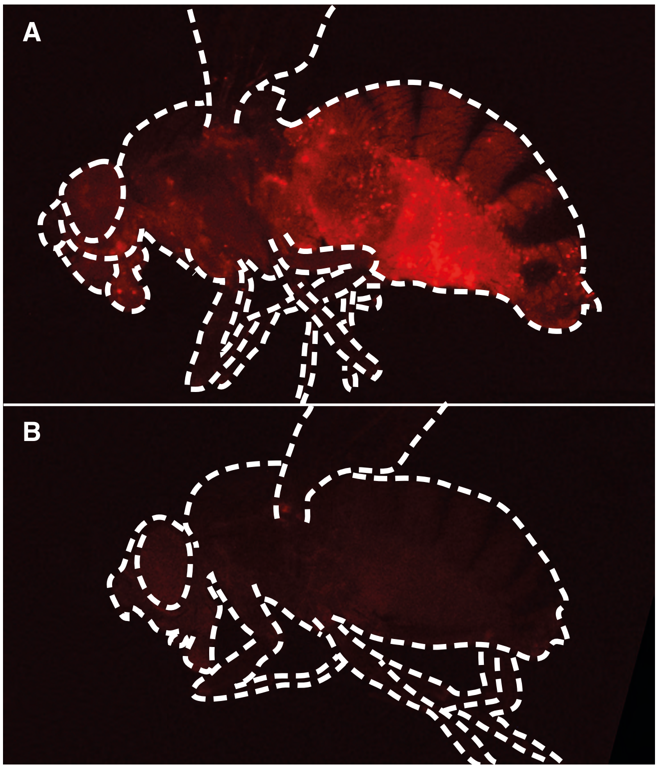

Figure S1. C virus infection, detected in live flies by a C virus-specific Munin-type reporter construct.

Drosophila C virus reporter flies were crossed to the UAS-RFP stock and C virus-infected cells were detected by fluorescence microscopy. (A) Fly infected by injection of C virus. (B) Uninjected control fly.

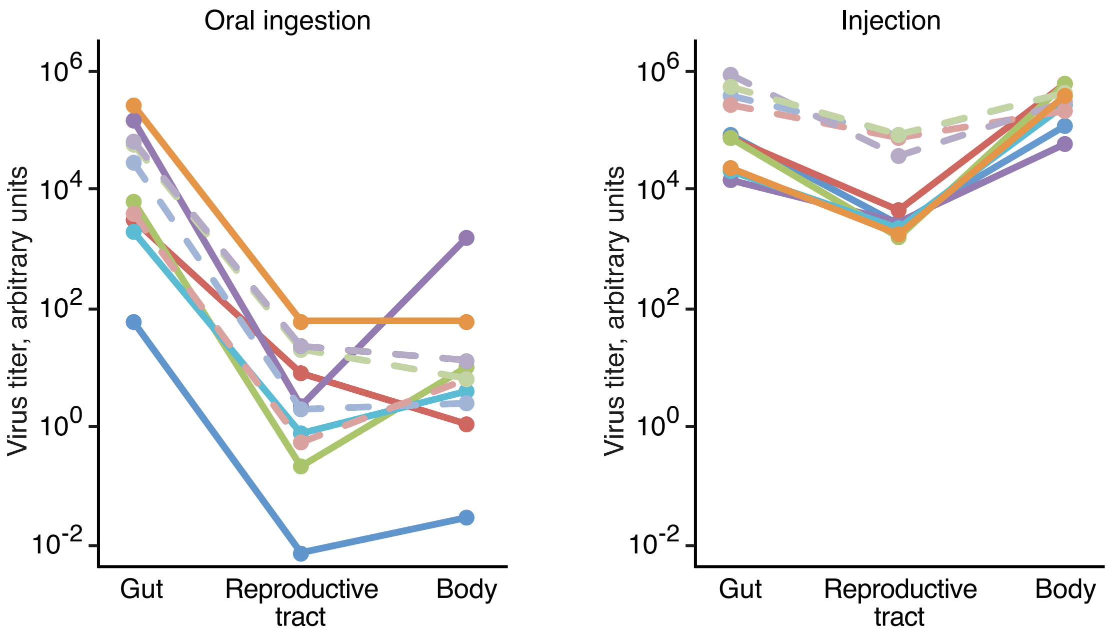

Figure S2. Viral titres for body parts of individual flies, determined by quantitative RT-PCR. Each line represents a single fly. Data are from two independent experiments (solid lines, experiment 1; dashed lines, experiment 2).

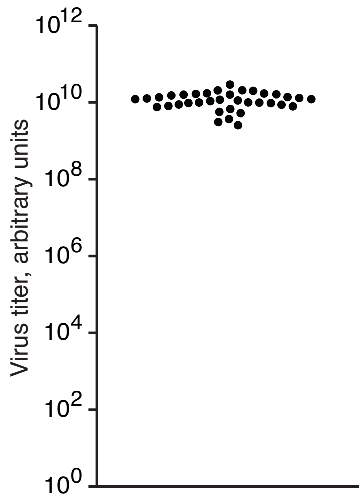

Figure S3. Total virus titres for flies injected with Nora virus four weeks after injection. All injected flies have an invariantly high virus titre, in contrast to flies infected by oral ingestion where the titres vary by more than six orders of magnitude between individual flies (Fig 1E, and References {Habayeb, 2009 #3;Habayeb, 2009 #4}). The whole flies were homogenized and the amount of Nora virus RNA was determined by quantitative RT-PCR. Each data point represents a single fly and the graph includes the data from two independent experiments.

Appendix

Complete sequence of the Nora virus Munin reporter transformation plasmid.

The first 3861 bp (indicated by colours) were constructed; the remaining part is the pUASTattB transformation vector (Bischof et al, Proc Natl Acad Sci U S A, 2007. 104:3312-7) excluding the UAS promoter.

Red font: Restriction endonuclease site.
Gray background: Alpha-tubulin 84B promoter.
Green background: Start codon.
Yellow background: First two transmembrane domains of *late bloomer.*
Red background: Linker encoding the Nora virus VP4B-VP4C junction with a site for the viral protease.
Cyan background: Gal4.

```
GCTAGCGGATCCGGCGCGCCTTCGAGTCCCAACTAGTCCTGCAAAATACCGCAGCAAGTTTTAGAGAGA
CCAAGTGCCATTACCTCTCCCACTTCAGTTATCGGTTATGCGGCGTTTAAGTCGACAGCTTGCCGTCTC
TAGCTCCGGTGCCTATATAAAGCAGCCCGCTTTCCACATTTCATATTCGTTTTACGTTTGTCAAGCCTC
ATAGCCGGCAGTTCGAACGTATACGCTCTCTGAGTCAGACCTCGAAATCGTAGCTCTACGCAATTCTGT
GAATTTTCCTTGTCGCGTGTGAAACACTTCCAATAAAAACTCAATATGGTGAGTACTTTAAAAAAAAAT
CTAGTGAAATAATGCTGAAAAGAAATTTGTGTGGGCAAAATTCAATGGGCAAAAACGCGATGCGGCTTT
TTCTCAAAATGGCGGCCGGCCTGCGTTTTTTCCTCAAAAGTGATGACGTCATGCCTGTTTTTTTTTTTG
TTCGCAATGAGGAATGGCTCTTAAAATCTAGATAAAAAAAATATTCATTATTTCTATGCTGCTGGAACG
CTTCATTAATCTTAAAAATTCTAAATTCGGTTACCATGATACTTCGACGCATAACTGTAGATTTTGGAT
AGAATTAAAGAGAAAATGGCGAGAGAGTAAAATTCCGGCGTCGGCAAAGTAGAGCAAAAAAATCAGTAT
ACCATTTAGCTACCTCTCTCACTCGCACGCAGTGCCGGCTCAAGCTGGGCGCGGCTCTGCAATTATCGA
TTTTCTTGGGGTGTGTAACTAATCATCCGTTTTCCCTTCCTCCTCATCCACAGCGTGAACGTACGATGG
GTTGCGCCACGACCAGCGTGAGGATCGCCTCCATCGTTCTGAATGCCGTTTTAGGGTTTCTTGCTGCTG
GGGCCATCGGCTGGATAGCTTACAATGCGGACACGGAGACGGAGGAATTCGTAATAGCCGCTTACATCG
CGTGCTCGCTCATCCTGGTCTTTGCTCTGCTGGGCATCTTCGCGGCCATCCGGGAATCGGTGGTGCTGA
CTGCAGCTAGCCCTGACAATAATGTGGATACACGTCATTTTGAGCTCGTTGAAGCTTTGATGTATCACT
ACGATAGCCTCCGAATTCGAGGAGAAGAACAGAGTCTACCAGAAAATGCACCTAATGCAGTTTCAAACC
CTCAGCAGTTTATCACACCCGCAACCGCTCTAAGTGCCGAAGAATATAATGTGCGCGGCCGCATGAAGC
TACTGTCTTCTATCGAACAAGCATGCGATATTTGCCGACTTAAAAAGCTCAAGTGCTCCAAAGAAAAAC
CGAAGTGCGCCAAGTGTCTGAAGAACAACTGGGAGTGTCGCTACTCTCCCAAAACCAAAAGGTCTCCGC
TGACTAGGGCACATCTGACAGAAGTGGAATCAAGGCTAGAAAGACTGGAACAGCTATTTCTACTGATTT
TTCCTCGAGAAGACCTTGACATGATTTTGAAAATGGATTCTTTACAGGATATAAAAGCATTGTTAACAG
GATTATTTGTACAAGATAATGTGAATAAAGATGCCGTCACAGATAGATTGGCTTCAGTGGAGACTGATA
TGCCTCTAACATTGAGACAGCATAGAATAAGTGCGACATCATCATCGGAAGAGAGTAGTAACAAAGGTC
AAAGACAGTTGACTGTATCGATTGACTCGGCAGCTCATCATGATAACTCCACAATTCCGTTGGATTTTA
TGCCCAGGGATGCTCTTCATGGATTTGATTGGTCTGAAGAGGATGACATGTCGGATGGCTTGCCCTTCC
TGAAAACGGACCCCAACAATAATGGGTTCTTTGGCGACGGTCCTCTCTTATGTATTCTTCGATCTATTG
GCTTTAAACCGGAAAATTACACGAACTCTAACGTTAACAGGCTCCCGACCATGATTACGGATAGATACA
CGTTGGCTTCTAGATCCACAACATCCCGTTTACTTCAAAGTTATCTCAATAATTTTCACCCCTACTGCC
CTATCGTGCACTCACCGACGCTAATGATGTTGTATAATAACCAGATTGAAATCGCGTCGAAGGATCAAT
GGCAAATCCTTTTTAACTGCATATTAGCCATTGGAGCCTGGTGTATAGAGGGGGAATCTACTGATATAG
ATGTTTTTTACTATCAAAATGCTAAATCTCATTTGACGAGCAAGGTCTTCGAGTCAGGTTCCATAATTT
TGGTGACAGCCCTACATCTTCTGTCGCGATATACACAGTGGAGGCAGAAAACAAATACTAGCTATAATT
TTCACAGCTTTTCCATAAGAATGGCCATATCATTGGGCTTGAATAGGGACCTCCCCTCGTCCTTCAGTG
ATAGCAGCATTCTGGAACAAAGACGCCGAATTTGGTGGTCTGTCTACTCTTGGGAGATCCAATTGTCCC
TGCTTTATGGTCGATCCATCCAGCTTTCTCAGAATACAATCTCCTTCCCTTCTTCTGTCGACGATGTGC
AGCGTACCACAACAGGTCCCACCATATATCATGGCATCATTGAAACAGCAAGGCTCTTACAAGTTTTCA
CAAAAATCTATGAACTAGACAAAACAGTAACTGCAGAAAAAAGTCCTATATGTGCAAAAAAATGCTTGA
TGATTTGTAATGAGATTGAGGAGGTTTCGAGACAGGCACCAAAGTTTTTACAAATGGATATTTCCACCA
CCGCTCTAACCAATTTGTTGAAGGAACACCCTTGGCTATCCTTTACAAGATTCGAACTGAAGTGGAAAC
AGTTGTCTCTTATCATTTATGTATTAAGAGATTTTTTCACTAATTTTACCCAGAAAAAGTCACAACTAG
AACAGGATCAAAATGATCATCAAAGTTATGAAGTTAAACGATGCTCCATCATGTTAAGCGATGCAGCAC
```

AAAGAACTGTTATGTCTGTAAGTAGCTATATGGACAATCATAATGTCACCCCATATTTTGCCTGGAATT
GTTCTTATTACTTGTTCAATGCAGTCCTAGTACCCATAAAGACTCTACTCTCAAACTCAAAATCGAATG
CTGAGAATAACGAGACCGCACAATTATTACAACAAATTAACACTGTTCTGATGCTATTAAAAAAACTGG
CCACTTTTAAAATCCAGACTTGTGAAAAATACATTCAAGTACTGGAAGAGGTATGTGCGCCGTTTCTGT
TATCACAGTGTGCAATCCCATTACCGCATATCAGTTATAACAATAGTAATGGTAGCGCCATTAAAAATA
TTGTCGGTTCTGCAACTATCGCCCAATACCCTACTCTTCCGGAGGAAAATGTCAACAATATCAGTGTTA
AATATGTTTCTCCTGGCTCAGTAGGGCCTTCACCTGTGCCATTGAAATCAGGAGCAAGTTTCAGTGATC
TAGTCAAGCTGTTATCTAACCGTCCACCCTCTCGTAACTCTCCAGTGACAATACCAAGAAGCACACCTT
CGCATCGCTCAGTCACGCCTTTTCTAGGGCAACAGCAACAGCTGCAATCATTAGTGCCACTGACCCCGT
CTGCTTTGTTTGGTGGCGCCAATTTTAATCAAAGTGGGAATATTGCTGATAGCTCATTGTCCTTCACTT
TCACTAACAGTAGCAACGGTCCGAACCTCATAACAACTCAAACAAATTCTCAAGCGCTTTCACAACCAA
TTGCCTCCTCTAACGTTCATGATAACTTCATGAATAATGAAATCACGGCTAGTAAAATTGATGATGGTA
ATAATTCAAAACCACTGTCACCTGGTTGGACGGACCAAACTGCGTATAACGCGTTTGGAATCACTACAG
GGATGTTTAATACCACTACAATGGATGATGTATATAACTATCTATTCGATGATGAAGATACCCCACCAA
ACCCAAAAAAAGAGTAGCCTAAGGATCTTTGTGAAGGAACCTTACTTCTGTGGTGTGACATAATTGGAC
AAACTACCTACAGAGATTTAAAGCTCTAAGGTAAATATAAAATTTTTAAGTGTATAATGTGTTAAACTA
CTGATTCTAATTGTTTGTGTATTTTAGATTCCAACCTATGGAACTGATGAATGGGAGCAGTGGTGGAAT
GCCTTTAATGAGGAAAACCTGTTTTGCTCAGAAGAAATGCCATCTAGTGATGATGAGGCTACTGCTGAC
TCTCAACATTCTACTCCTCCAAAAAAGAAGAGAAAGGTAGAAGACCCCAAGGACTTTCCTTCAGAATTG
CTAAGTTTTTTGAGTCATGCTGTGTTTAGTAATAGAACTCTTGCTTGCTTTGCTATTTACACCACAAAG
GAAAAAGCTGCACTGCTATACAAGAAAATTATGGAAAAATATTTGATGTATAGTGCCTTGACTAGAGAT
CATAATCAGCCATACCACATTTGTAGAGGTTTTACTTGCTTTAAAAAACCTCCCACACCTCCCCCTGAA
CCTGAAACATAAAATGAATGCAATTGTTGTTGTTAACTTGTTTATTGCAGCTTATAATGGTTACAAATA
AAGCAATAGCATCACAAATTTCACAAATAAAGCATTTTTTTCACTGCATTCTAGTTGTGGTTTGTCCAA
ACTCATCAATGTATCTTATCATGTCTGGATCCACTAGTGTCGACGATGTAGGTCACGGTCTCGAAGCCG
CGGTGCGGGTGCCAGGGCGTGCCCTTGGGCTCCCCGGGCGCGTACTCCACCTCACCCATCTGGTCCATC
ATGATGAACGGGTCGAGGTGGCGGTAGTTGATCCCGGCGAACGCGCGGCGCACCGGGAAGCCCTCGCCC
TCGAAACCGCTGGGCGCGGTGGTCACGGTGAGCACGGGACGTGCGACGGCGTCGGCGGGTGCGGATACG
CGGGGCAGCGTCAGCGGGTTCTCGACGGTCACGGCGGGCATGTCGACACTAGTTCTAGCCAGCTTTTGT
TCCCTTTAGTGAGGGTTAATTTCGAGCTTGGCGTAATCATGGTCATAGCTGTTTCCTGTGTGAAATTGT
TATCCGCTCACAATTCCACACAACATACGAGCCGGAAGCATAAAGTGTAAAGCCTGGGGTGCCTAATGA
GTGAGCTAACTCACATTAATTGCGTTGCGCTCACTGCCCGCTTTCCAGTCGGGAAACCTGTCGTGCCAG
CTGCATTAATGAATCGGCCAACGCGCGGGGAGAGGCGGTTTGCGTATTGGGCGCTCTTCCGCTTCCTCG
CTCACTGACTCGCTGCGCTCGGTCGTTCGGCTGCGGCGAGCGGTATCAGCTCACTCAAAGGCGGTAATA
CGGTTATCCACAGAATCAGGGGATAACGCAGGAAAGAACATGTGAGCAAAAGGCCAGCAAAAGGCCAGG
AACCGTAAAAAGGCCGCGTTGCTGGCGTTTTTCCATAGGCTCCGCCCCCCTGACGAGCATCACAAAAAT
CGACGCTCAAGTCAGAGGTGGCGAAACCCGACAGGACTATAAAGATACCAGGCGTTTCCCCCTGGAAGC
TCCCTCGTGCGCTCTCCTGTTCCGACCCTGCCGCTTACCGGATACCTGTCCGCCTTTCTCCCTTCGGGA
AGCGTGGCGCTTTCTCATAGCTCACGCTGTAGGTATCTCAGTTCGGTGTAGGTCGTTCGCTCCAAGCTG
GGCTGTGTGCACGAACCCCCCGTTCAGCCCGACCGCTGCGCCTTATCCGGTAACTATCGTCTTGAGTCC
AACCCGGTAAGACACGACTTATCGCCACTGGCAGCAGCCACTGGTAACAGGATTAGCAGAGCGAGGTAT
GTAGGCGGTGCTACAGAGTTCTTGAAGTGGTGGCCTAACTACGGCTACACTAGAAGAACAGTATTTGGT
ATCTGCGCTCTGCTGAAGCCAGTTACCTTCGGAAAAAGAGTTGGTAGCTCTTGATCCGGCAAACAAACC
ACCGCTGGTAGCGGTGGTTTTTTTGTTTGCAAGCAGCAGATTACGCGCAGAAAAAAAGGATCTCAAGAA
GATCCTTTGATCTTTTCTACGGGGTCTGACGCTCAGTGGAACGAAAACTCACGTTAAGGGATTTTGGTC
ATGAGATTATCAAAAAGGATCTTCACCTAGATCCTTTTAAATTAAAAATGAAGTTTTAAATCAATCTAA
AGTATATATGAGTAAACTTGGTCTGACAGTTACCAATGCTTAATCAGTGAGGCACCTATCTCAGCGATC
TGTCTATTTCGTTCATCCATAGTTGCCTGACTCCCCGTCGTGTAGATAACTACGATACGGGAGGGCTTA
CCATCTGGCCCCAGTGCTGCAATGATACCGCGAGACCCACGCTCACCGGCTCCAGATTTATCAGCAATA
AACCAGCCAGCCGGAAGGGCCGAGCGCAGAAGTGGTCCTGCAACTTTATCCGCCTCCATCCAGTCTATT
AATTGTTGCCGGGAAGCTAGAGTAAGTAGTTCGCCAGTTAATAGTTTGCGCAACGTTGTTGCCATTGCT
ACAGGCATCGTGGTGTCACGCTCGTCGTTTGGTATGGCTTCATTCAGCTCCGGTTCCCAACGATCAAGG
CGAGTTACATGATCCCCCATGTTGTGCAAAAAAGCGGTTAGCTCCTTCGGTCCTCCGATCGTTGTCAGA
AGTAAGTTGGCCGCAGTGTTATCACTCATGGTTATGGCAGCACTGCATAATTCTCTTACTGTCATGCCA
TCCGTAAGATGCTTTTCTGTGACTGGTGAGTACTCAACCAAGTCATTCTGAGAATAGTGTATGCGGCGA
CCGAGTTGCTCTTGCCCGGCGTCAATACGGGATAATACCGCGCCACATAGCAGAACTTTAAAAGTGCTC
ATCATTGGAAAACGTTCTTCGGGGCGAAAACTCTCAAGGATCTTACCGCTGTTGAGATCCAGTTCGATG
TAACCCACTCGTGCACCCAACTGATCTTCAGCATCTTTTACTTTCACCAGCGTTTCTGGGTGAGCAAAA
ACAGGAAGGCAAAATGCCGCAAAAAAGGGAATAAGGGCGACACGGAAATGTTGAATACTCATACTCTTC
CTTTTTCAATATTATTGAAGCATTTATCAGGGTTATTGTCTCATGAGCGGATACATATTTGAATGTATT
TAGAAAAATAAACAAATAGGGGTTCCGCGCACATTTCCCCGAAAAGTGCCACCTAAATTGTAAGCGTTA
ATATTTTGTTAAAATTCGCGTTAAATTTTTGTTAAATCAGCTCATTTTTTAACCAATAGGCCGAAATCG
GCAAAATCCCTTATAAATCAAAAGAATAGACCGAGATAGGGTTGAGTGTTGTTCCAGTTTGGAACAAGA

GTCCACTATTAAAGAACGTGGACTCCAACGTCAAAGGGCGAAAAACCGTCTATCAGGGCGATGGCCCAC
TACGTGAACCATCACCCTAATCAAGTTTTTTGGGGTCGAGGTGCCGTAAAGCACTAAATCGGAACCCTA
AAGGGAGCCCCCGATTTAGAGCTTGACGGGGAAAGCCGGCGAACGTGGCGAGAAAGGAAGGGAAGAAAG
CGAAAGGAGCGGGCGCTAGGGCGCTGGCAAGTGTAGCGGTCACGCTGCGCGTAACCACCACACCCGCCG
CGCTTAATGCGCCGCTACAGGGCGCGTCCCATTCGCCATTCAGGCTGCGCAACTGTTGGGAAGGGCGAT
CGGTGCGGGCCTCTTCGCTATTACGCCAGCTGGCGAAAGGGGGATGTGCTGCAAGGCGATTAAGTTGGG
TAACGCCAGGGTTTTCCCAGTCACGACGTTGTAAAACGACGGCCAGTGAATTGTAATACGACTCACTAT
AGGGCGAATTGGGTACGTACCGGGCCCCCTAGTATGTATGTAAGTTAATAAAACCCATTTTTGCGGAAAG
TAGATAAAAAAAACATTTTTTTTTTTTACTGCACTGGATATCATTGAACTTATCTGATCAGTTTTAAAT
TTACTTCGATCCAAGGGTATTTGATGTACCAGGTTCTTTCGATTACCTCTCACTCAAAATGACATTCCA
CTCAAAGTCAGCGCTGTTTGCCTCCTTCTCTGTCCACAGAAATATCGCCGTCTCTTTCGCCGCTGCGTC
CGCTATCTCTTTCGCCACCGTTTGTAGCGTTACGTAGCGTCAATGTCCGCCTTCAGTTGCATTTTGTCA
GCGGTTTCGTGACGAAGCTCCAAGCGGTTTACGCCATCAATTAAACACAAAGTGCTGTGCCAAAACTCC
TCTCGCTTCTTATTTTTGTTTGTTTTTTGAGTGATTGGGGTGGTGATTGGTTTTGGGTGGGTAAGCAGG
GGAAAGTGTGAAAAATCCCGGCAATGGGCCAAGAGGATCAGGAGCTATTAATTCGCGGAGGCAGCAAAC
ACCCATCTGCCGAGCATCTGAACAATGTGAGTAGTACATGTGCATACATCTTAAGTTCACTTGATCTAT
AGGAACTGCGATTGCAACATCAAATTGTCTGCGGCGTGAGAACTGCGACCCACAAAAATCCCAAACCGC
AATTGCACAAACAAATAGTGACACGAAACAGATTATTCTGGTAGCTGTTCTCGCTATATAAGACAATTT
TTGAGATCATATCATGATCAAGACATCTAAAGGCATTCATTTTCGACTATATTCTTTTTTACAAAAAAT
ATAACAACCAGATATTTTAAGCTGATCCTAGATGCACAAAAAATAAATAAAAGTATAAACCTACTTCGT
AGGATACTTCGGGGTACTTTTTGTTCGGGGTTAGATGAGCATAACGCTTGTAGTTGATATTTGAGATCC
CCTATCATTGCAGGGTGACAGCGGAGCGGCTTCGCAGAGCTGCATTAACCAGGGCTTCGGGCAGGCCAA
AAACTACGGCACGCTCCGGCCACCCAGTCCGCCGGAGGACTCCGGTTCAGGGAGCGGCCAACTAGCCGA
GAACCTCACCTATGCCTGGCACAATATGGACATCTTTGGGGCGGTCAATCAGCCGGGCTCCGGATGGCG
GCAGCTGGTCAACCGGACACGCGGACTATTCTGCAACGAGCGACACATACCGGCGCCCAGGAAACATTT
GCTCAAGAACGGTGAGTTTCTATTCGCAGTCGGCTGATCTGTGTGAAATCTTAATAAAGGGTCCAATTA
CCAATTTGAAACTCAGTTTGCGGCGTGGCCTATCCGGGCGAACTTTTGGCCGTGATGGGCAGTTCCGGT
GCCGGAAAGACGACCCTGCTGAATGCCCTTGCCTTTCGATCGCCGCAGGGCATCCAAGTATCGCCATCC
GGGATGCGACTGCTCAATGGCCAACCTGTGGACGCCAAGGAGATGCAGGCCAGGTGCGCCTATGTCCAG
CAGGATGACCTCTTTATCGGCTCCCTAACGGCCAGGGAACACCTGATTTTCCAGGCCATGGTGCGGATG
CCACGACATCTGACCTATCGGCAGCGAGTGGCCCGCGTGGATCAGGTGATCCAGGAGCTTTCGCTCAGC
AAATGTCAGCACACGATCATCGGTGTGCCCGGCAGGGTGAAAGGTCTGTCCGGCGGAGAAAGGAAGCGT
CTGGCATTCGCCTCCGAGGCACTAACCGATCCGCCGCTTCTGATCTGCGATGAGCCCACCTCCGGACTG
GACTCATTTACCGCCCACAGCGTCGTCCAGGTGCTGAAGAAGCTGTCGCAGAAGGGCAAGACCGTCATC
CTGACCATTCATCAGCCGTCTTCCGAGCTGTTTGAGCTCTTTGACAAGATCCTTCTGATGGCCGAGGGC
AGGGTAGCTTTCTTGGGCACTCCCAGCGAAGCCGTCGACTTCTTTTCCTAGTGAGTTCGATGTGTTTAT
TAAGGGTATCTAGCATTACATTACATCTCAACTCCTATCCAGCGTGGGTGCCCAGTGTCCTACCAACTA
CAATCCGGCGGACTTTTACGTACAGGTGTTGGCCGTTGTGCCCGGACGGGAGATCGAGTCCCGTGATCG
GATCGCCAAGATATGCGACAATTTTGCTATTAGCAAAGTAGCCCGGGATATGGAGCAGTTGTTGGCCAC
CAAAAATTTGGAGAAGCCACTGGAGCAGCCGGAGAATGGGTACACCTACAAGGCCACCTGGTTCATGCA
GTTCCGGGCGGTCCTGTGGCGATCCTGGCTGTCGGTGCTCAAGGAACCACTCCTCGTAAAAGTGCGACT
TATTCAGACAACGGTGAGTGGTTCCAGTGGAAACAAATGATATAACGCTTACAATTCTTGGAAACAAAT
TCGCTAGATTTTAGTTAGAATTGCCTGATTCCACACCCTTCTTAGTTTTTTTCAATGAGATGTATAGTT
TATAGTTTTGCAGAAAATAAATAAATTTCATTTAACTCGCGAACATGTTGAAGATATGAATATTAATGA
GATGCGAGTAACATTTTAATTTGCAGATGGTTGCCATCTTGATTGGCCTCATCTTTTTGGGCCAACAAC
TCACGCAAGTGGGCGTGATGAATATCAACGGAGCCATCTTCCTCTTCCTGACCAACATGACCTTTCAAA
ACGTCTTTGCCACGATAAATGTAAGTCTTGTTTAGAATACATTTGCATATTAATAATTTACTAACTTTC
TAATGAATCGATTCGATTTAGGTGTTCACCTCAGAGCTGCCAGTTTTTATGAGGGAGGCCCGAAGTCGA
CTTTATCGCTGTGACACATACTTTCTGGGCAAAACGATTGCCGAATTACCGCTTTTTCTCACAGTGCCA
CTGGTCTTCACGGCGATTGCCTATCCGATGATCGGACTGCGGGCCGGAGTGCTGCACTTCTTCAACTGC
CTGGCGCTGGTCACTCTGGTGGCCAATGTGTCAACGTCCTTCGGATATCTAATATCCTGCGCCAGCTCC
TCGACCTCGATGGCGCTGTCTGTGGGTCCGCCGGTTATCATACCATTCCTGCTCTTTGGCGGCTTCTTC
TTGAACTCGGGCTCGGTGCCAGTATACCTCAAATGGTTGTCGTACCTCTCATGGTTCCGTTACGCCAAC
GAGGGTCTGCTGATTAACCAATGGGCGGACGTGGAGCCGGGCGAAATTAGCTGCACATCGTCGAACACC
ACGTGCCCCAGTTCGGGCAAGGTCATCCTGGAGACGCTTAACTTCTCCGCCGCCGATCTGCCGCTGGAC
TACGTGGGTCTGGCCATTCTCATCGTGAGCTTCCGGGTGCTCGCATATCTGGCTCTAAGACTTCGGGCC
CGACGCAAGGAGTAGCCGACATATATCCGAAATAACTGCTTGTTTTTTTTTTTTACCATTATTACCATC
GTGTTTACTGTTTATTGCCCCCTCAAAAAGCTAATGTAATTATATTTGTGCCAATAAAAACAAGATATG
ACCTATAGAATACAAGTATTTCCCCTTCGAACATCCCCACAAGTAGACTTTGGATTTGTCTTCTAACCA
AAAGACTTACACACCTGCATACCTTACATCAAAAACTCGTTTATCGCTACATAAAACACCGGGATATAT
TTTTTATATACATACTTTTCAAATCGCGCGCCCTCTTCATAATTCACCTCCACCACACCACGTTTCGTA
GTTGCTCTTTCGCTGTCTCCCACCCGCTCTCCGCAACACATTCACCTTTTGTTCGACGACCTTGGAGCG
ACTGTCGTTAGTTCCGCGCGATTCGGTTCGCTCAAATGGTTCCGAGTGGTTCATTTCGTCTCAATAGAA

ATTAGTAATAAATATTTGTATGTACAATTTATTTGCTCCAATATATTTGTATATATTTCCCTCACAGCT
ATATTTATTCTAATTTAATATTATGACTTTTTAAGGTAATTTTTTGTGACCTGTTCGGAGTGATTAGCG
TTACAATTTGAACTGAAAGTGACATCCAGTGTTTGTTCCTTGTGTAGATGCATCTCAAAAAAATGGTGG
GCATAATAGTGTTGTTTATATATATCAAAAATAACAACTATAATAATAAGAATACATTTAATTTAGAAA
ATGCTTGGATTTCACTGGAACTAGGCTAGCATAACTTCGTATAATGTATGCTATACGAAGTTAT

Unique part of the C virus reporter sequence.

For this reporter, the virus protease cleavage site is encoded by:

GCTAGCCAGCGTGAGGAGGAGTATGACAACAACATACCTCAAACTATTTCTAATCGTGGGAAACGTGAG
GTTGAAGATGCTCGTATTGTTGCGCAAGTGATGGGTGAGGATGTAGCTATTCAAAGAAACGATGCTCAA
CATGGTGTTCATCCAATGACCATAGACACTCATAAGATCGACTCAAATTGCGGCCGC
